# Author Correction: KDM3 epigenetically controls tumorigenic potentials of human colorectal cancer stem cells through Wnt/β-catenin signalling

**DOI:** 10.1038/s41467-019-12878-z

**Published:** 2019-11-04

**Authors:** Jiong Li, Bo Yu, Peng Deng, Yingduan Cheng, Yongxin Yu, Kareena Kevork, Sivakumar Ramadoss, Xiangming Ding, Xinmin Li, Cun-Yu Wang

**Affiliations:** 1 Laboratory of Molecular Signaling, Division of Oral Biology and Medicine, School of Dentistry and Broad Stem Cell Research Center, UCLA, Los Angeles, California 90095 USA; 2 Department of Pathology and Laboratory Medicine, David Geffen School of Medicine, UCLA, Los Angeles, California 90095 USA; 3 Department of Bioengineering, Henry Samueli School of Engineering and Applied Science, UCLA, Los Angeles, California 90095 USA

Correction to: *Nature Communications* 10.1038/ncomms15146, published online 25 April 2017.

This Article contains errors in Figure 4 and Supplementary Figure 3. In Figure 4b, the image representing ALDH^−^/EpCAM^+^ Control siRNA was taken from the ALDH^+^/EpCAM^+^ KDM3A+B siRNA image. In Supplementary Figure 3b, the image representing CD133^+^/EpCAM^+^ was taken from CD133^+^/EpCAM^+^ Control siRNA image of panel a. The correct versions of Figure 4 and Supplementary Figure 3 are shown below. The error has not been corrected in the PDF or HTML versions of the Article.

**Figure 1:**
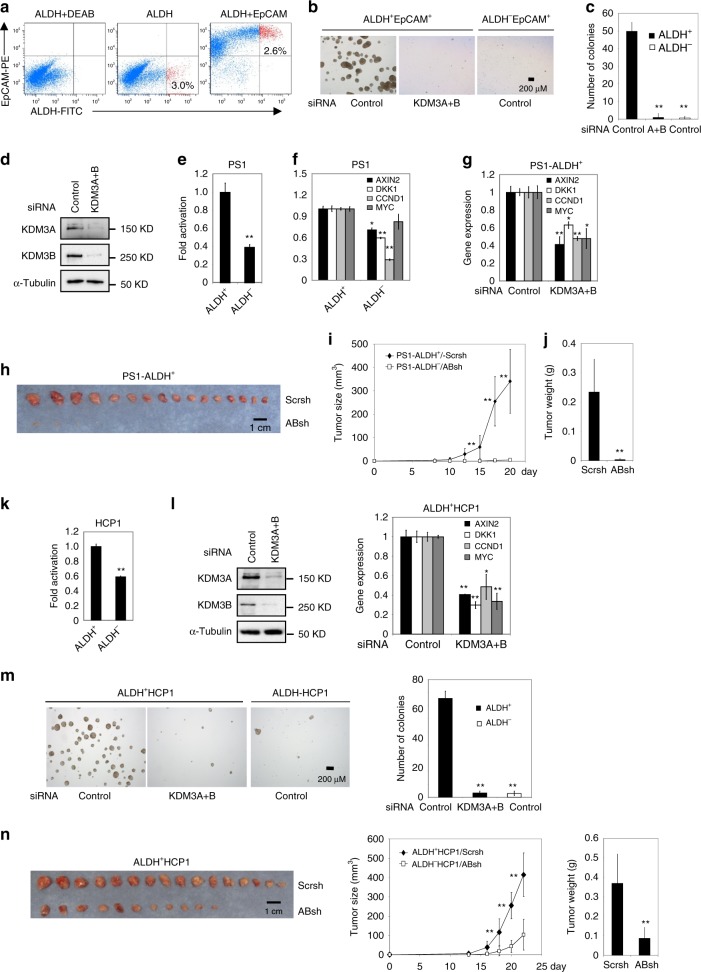
Fig. 4

**Figure 2:**
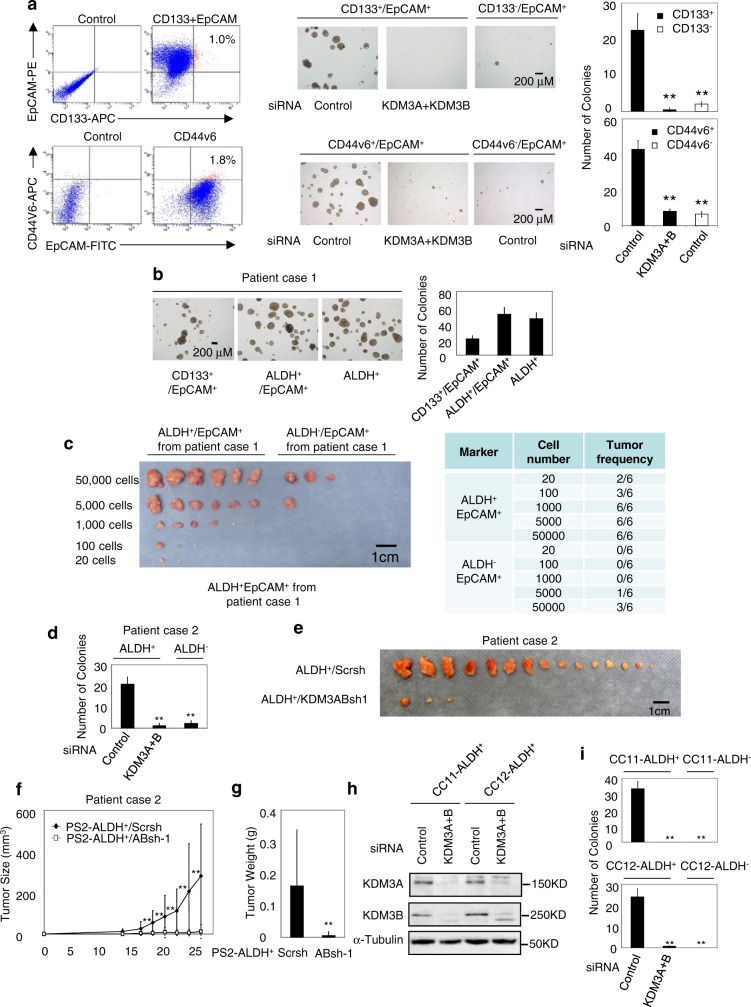
Supplementary Fig. 3

