# Supplementary material for: KDM3 epigenetically controls tumorigenic potentials of human colorectal cancer stem cells through Wnt/β-catenin signalling
Source: Nat Commun. 2017 Apr 25;8:15146. doi: 10.1038/ncomms15146 (PMC5414094; doi:10.1038/ncomms15146)
Supplement: Supplementary Information — Supplementary Figures and Supplementary Tables [file ncomms15146-s1.pdf]

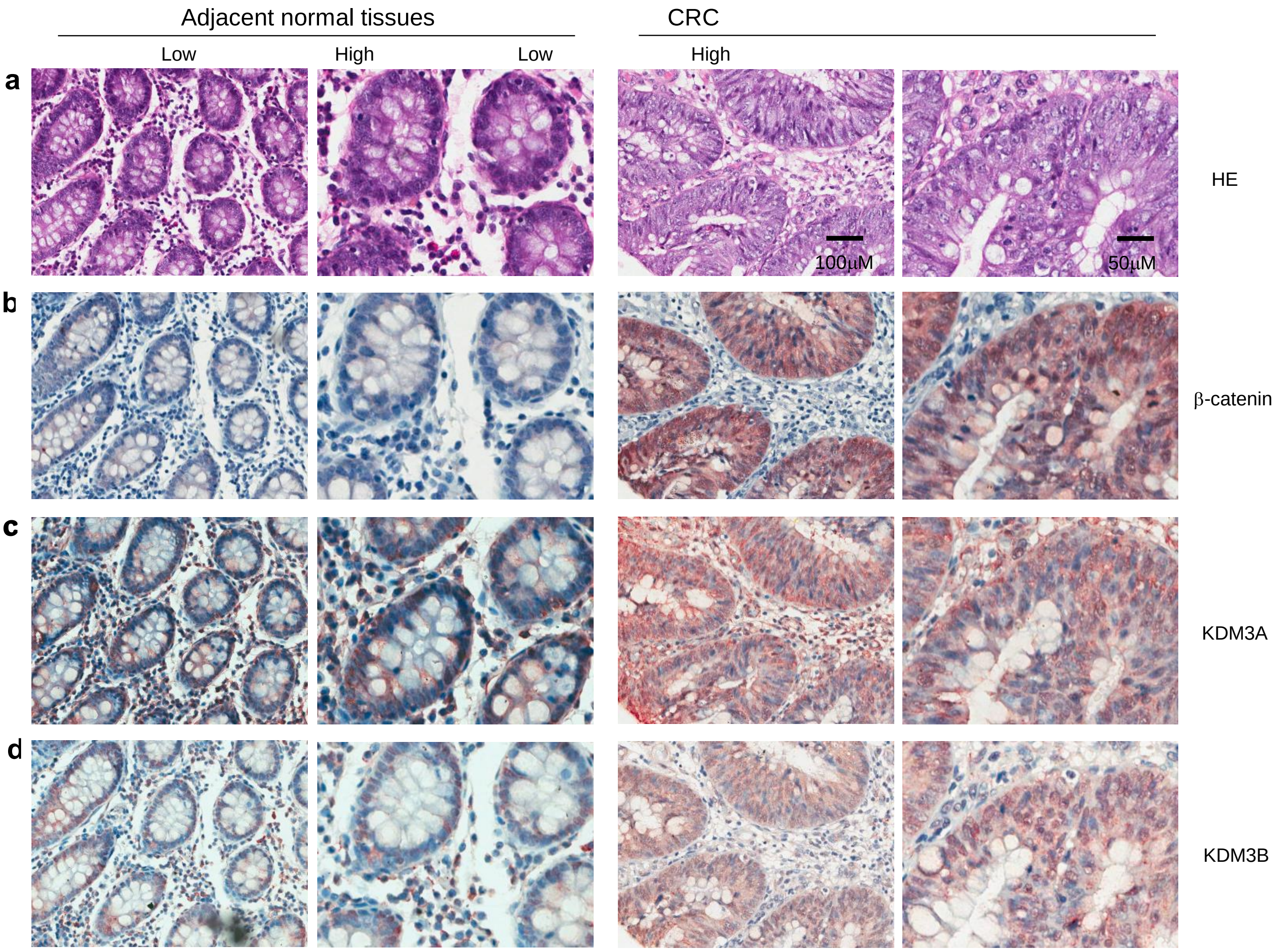

Supplementary Figure 1. The expression of KDM3A and KDM3B is upregulated in human CRCs. (a) The HE staining of representative tumor and adjacent normal tissues in TMA. **(b-d)** Representative immunostaining of β-catenin, KDM3A and KDM3B in tumor and adjacent normal tissues of TMA.

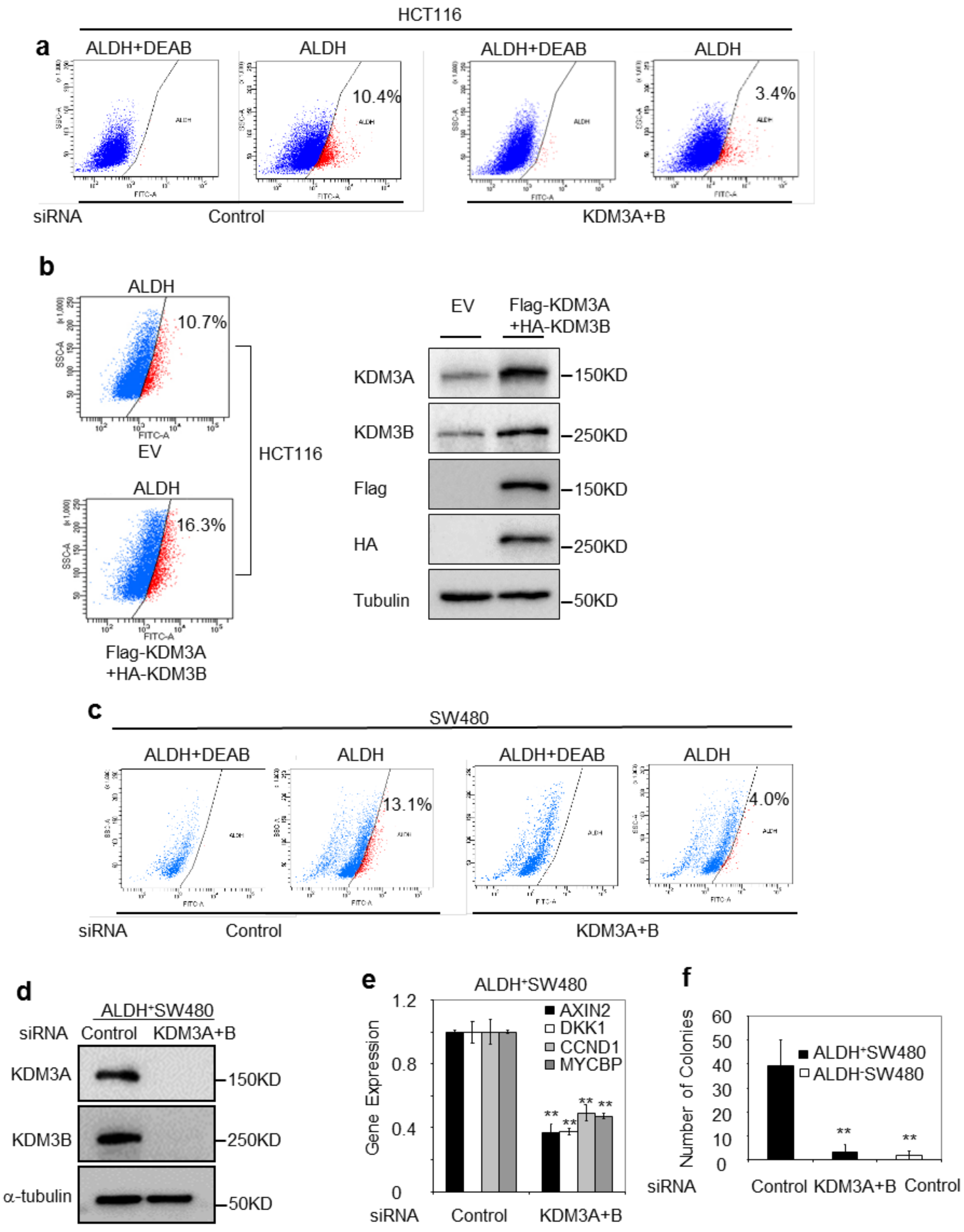

Supplementary Figure 2. KDM3A/B control the tumorigenic Potential of CSC-like cells isolated from human colorectal cancer cells. a. The percentage of ALDH^{+} subpopulations in HCT116 cells was reduced by knockdown of KDM3A/B, as determined by flow cytometry. **b.** The percentage of ALDH^{+} subpopulations in HCT116 cells was increased by expression of KDM3A/B, as determined by flow cytometry. **c.** The knockdown of KDM3A/B reduced the percentage of ALDH^{+} subpopulations in SW480 cells. **d**. The knockdown of KDM3A/B by siRNA in ALDH^{+}SW480 cells. **e.** The knockdown of KDM3A/B inhibited the expression of *AXIN2*, *DKK1*, *CCND1*, and *MYC* in ALDH^{+}SW480 cells. **f.** The knockdown of KDM3A/B inhibited tumorsphere formation of ALDH^{+}SW480 cells.

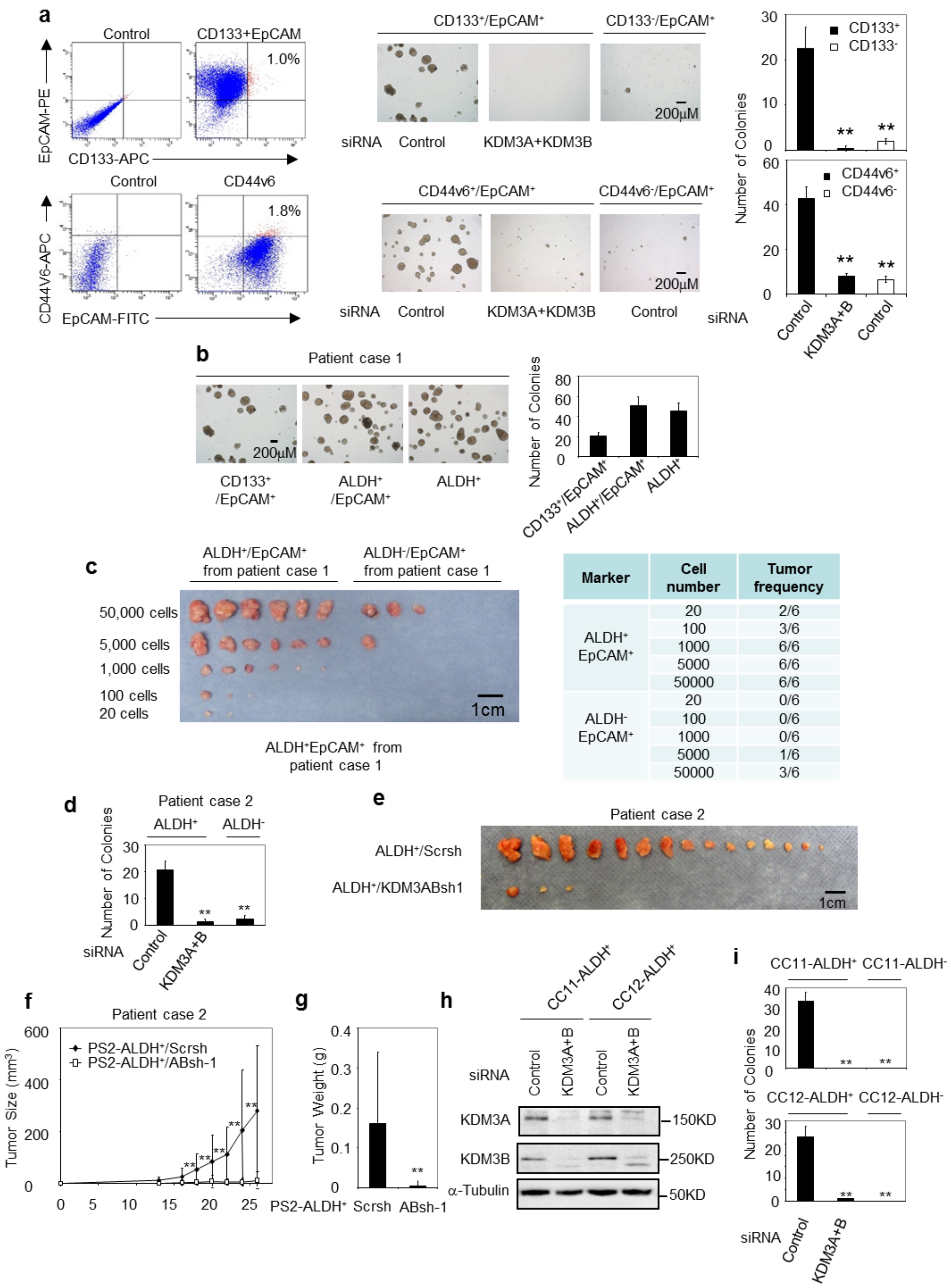

Supplementary Figure 3. KDM3A/B control tumorigenic potentials of human colorectal CSCs. a. Knockdown of KDM3A/B inhibited tumorsphere formation of CD133$^+$/EpCAM$^+$ and CD44v6$^+$EpCAM$^+$ cells isolated from patient case #1 (PS1). **b.** Tumorsphere formation assays of CD133$^+$/EpCAM$^+$, ALDH$^+$EpCAM$^+$ and ALDH$^+$ cells isolated from PS1. **c.** Tumor formation capacity of ALDH$^+$EpCAM$^+$ and ALDH$^-$EpCAM$^+$ cells in nude mice. The numbers of injected cells and the tumor frequencies are indicated. **d.** The knockdown of KDM3A/B inhibited tumorsphere formation of ALDH$^+$CSCs isolated from patient case #2 (PS2). **e-g.** The knockdown of KDM3A/B inhibited tumorigenic potentials of ALDH$^+$ EpCAM$^+$ cells isolated from PC2 in vivo. **P<0.01, unpaired 2-tailed Student's *t*-Test (n=16). **h.** The knockdown of KDM3A/B in ALDH$^+$CSCs isolated from CC-11 and CC-22 cells. **i.** The knockdown of KDM3A/B inhibited tumorsphere formation of ALDH$^+$CSCs isolated from CC-11 and CC-22 cells. **P<0.01, unpaired 2-tailed Student's *t*-Test (n=3).

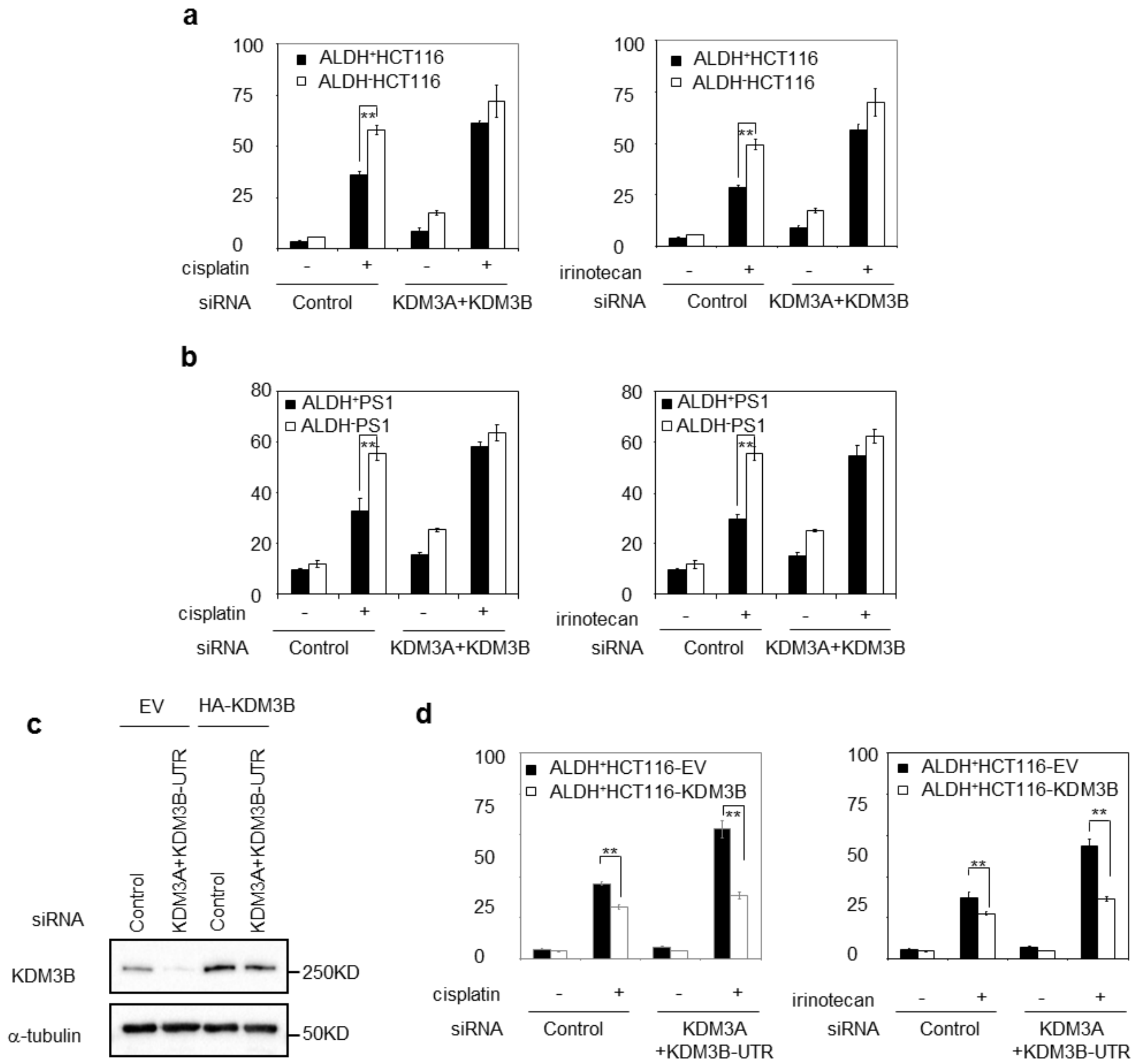

Supplementary Figure 4. KDM3A/B promote chemoresistance in human CRC CSCs. a. The knockdown of KDM3A/B enhanced cell death induced by cisplatin in ALDH$^+$HCT116 and ALDH$^-$HCT116 cells. **b.** The knockdown of KDM3A/B enhanced cell death induced by cisplatin in ALDH$^+$ and ALDH$^-$ cells from PS1. **c.** Western blot showed that overexpression of KDM3B in KDM3A/B knockdown ALDH$^+$HCT116 cells. d. Overexpression of KDM3B rescued cell death in KDM3A/B knockdown ALDH$^+$HCT116 cells induced by cisplatin or irinotecan. ***P*<0.01, unpaired 2-tailed Student's *t*-Test (n=3).

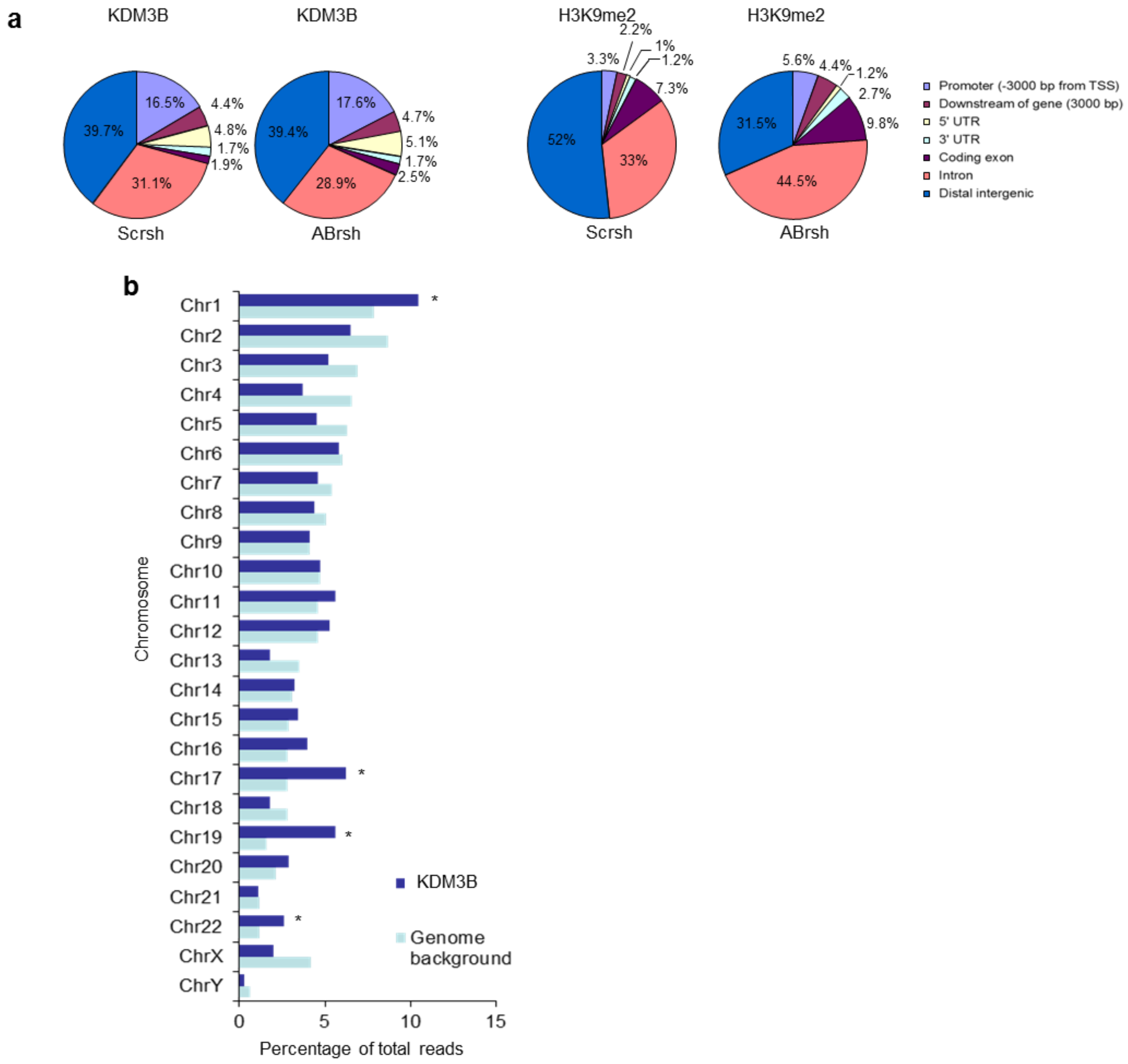

Supplementary Figure 5. The genome distribution of KDM3B and H3K9me2 enrichment in HCT116 cells. a. Pie graph of KDM3B and H3K9me2 occupancy in relation to gene structure following depletion of KDM3A/B. A promoter is defined as 3 kb upstream and downstream of the transcription start site. Intergenic regions are defined as at least 3kb from start and end of any transcript. **b.** Enrichment pattern of KDM3B-bound regions among individual chromosomes is shown as a bar chart. Percent of total KDM3B-binding sites and what would be expected by random chance for each chromosome is shown. The asterisks denotes enrichment *P*-value<10^{-4}.

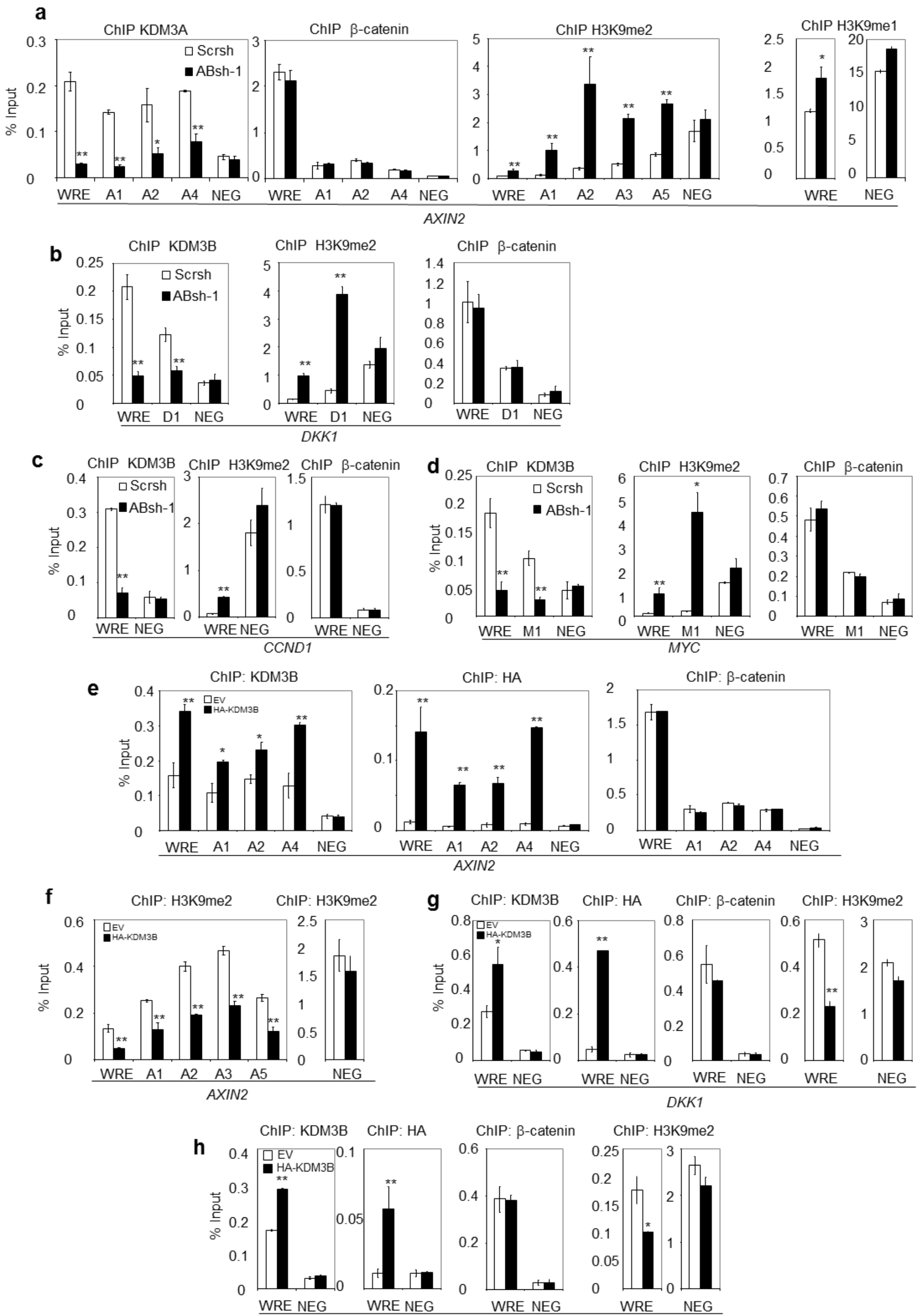

Supplementary Figure 6. KDM3B binds to Wnt target promoters and demethylates H3K9me2 marks. a. ChIP-qPCR analysis of the *AXIN2* promoter, either on WRE (Wnt-regulated enhancer), A1-A5 (introns and exons) or NEG (negative control) in HCT116/ABsh-1 cells and HCT116/Scrsh cells. **b.** ChIP-qPCR analysis of the *DKK1* promoter in HCT116/ABsh-1 cells and HCT116/Scrsh cells. **c.** ChIP-qPCR analysis of the *CCND1* promoter in HCT116/ABsh-1 cells and HCT116/Scrsh cells. **d.** ChIP-qPCR analysis of the *MYC* promoter in HCT116/ABsh-1 cells and HCT116/Scrsh cells. **e-h.** ChIP-qPCR analysis of the *AXIN2*, *DKK1* and *CCND1* promoters in HCT116 cells transfected with HA-tagged KDM4B expression vectors or control empty vectors (EV). HCT116 cells were transfected with HA-tagged KDM3B expression vectors (HA-KDM3B) or control empty vectors. *P<0.05, **P<0.01, unpaired 2-tailed student's t-Test (n=3).

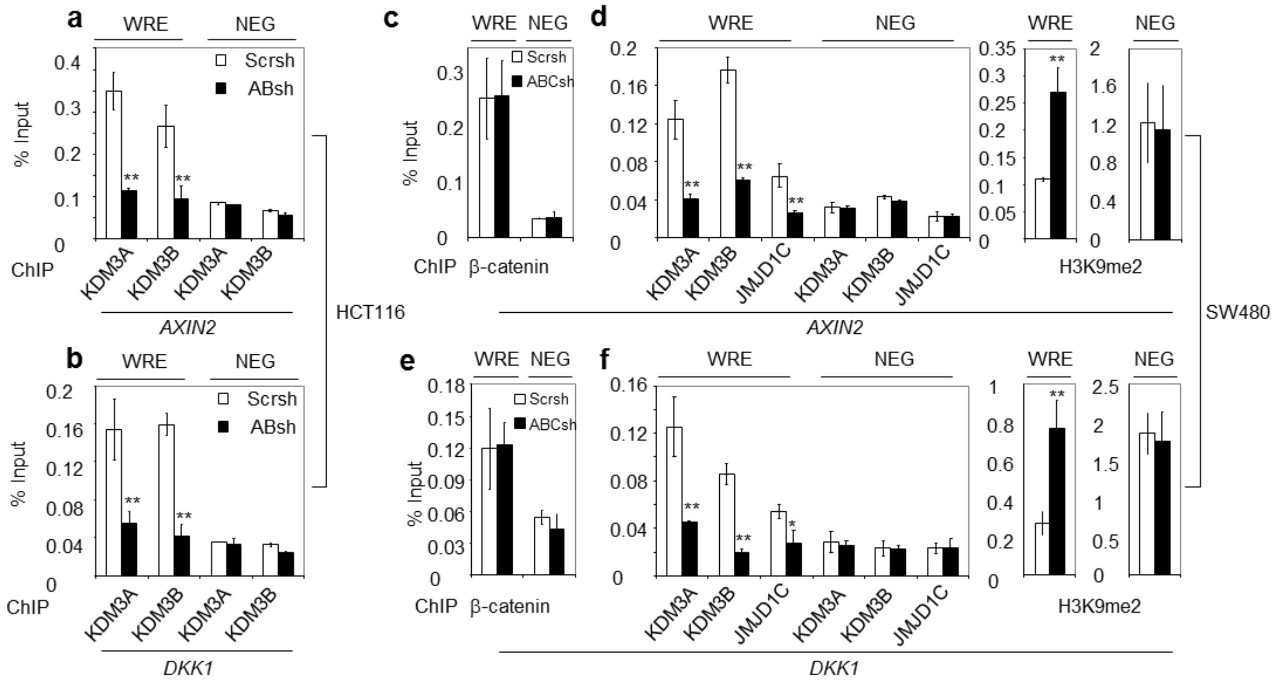

Supplementary Figure 7. KDM3A/B bind to Wnt target promoters and demethylate H3K9me2 marks. a. KDM3A/B bound to the promoter of *AXIN2* in HCT116 cells. **b.** KDM3A/B bound to the promoter of *DKK1* in HCT116 cells. **c.** The knockdown of KDM3A/B did not affect β-catenin binding to the promoter of *AXIN2* in SW480 cells. **d.** The knockdown of KDM3A/B increased the levels of H3K9me2 on the promoter of *AXIN2* in SW480 cells. **e.** The knockdown of KDM3A/B did not affect β-catenin binding to the promoter of *DKK1* in SW480 cells. **f.** The knockdown of KDM3A/B increased the levels of H3K9me2 on the promoter of *DKK1* in SW480 cells. **P*<0.05, ***P*<0.01, unpaired 2-tailed Student's *t*-Test (n=3).

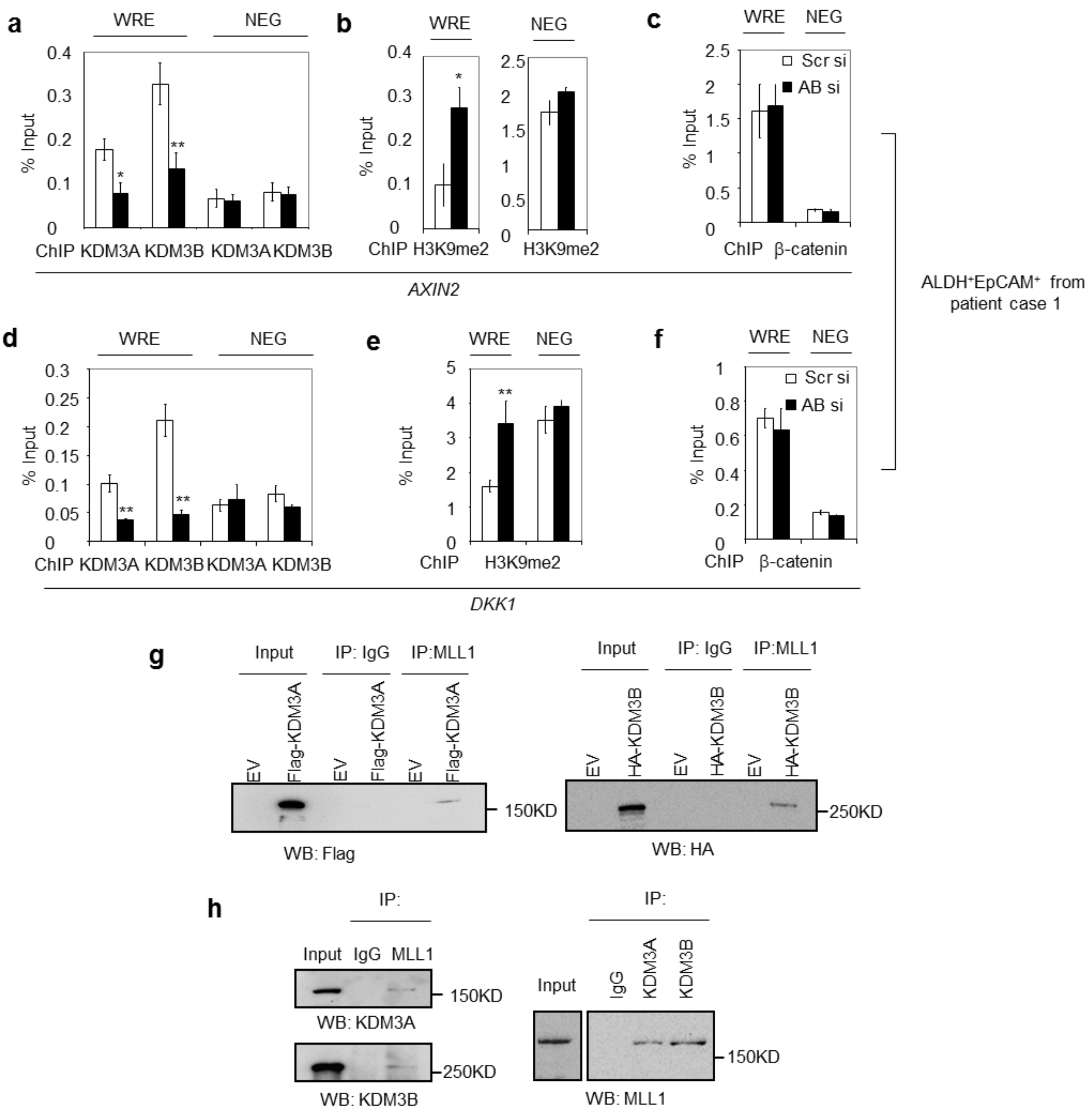

Supplementary Figure 8. KDM3A/B demethylate H3K9me2 on the Wnt target gene promoter in human colorectal CSCs. a. ChIP assays confirmed that the knockdown of KDM3A/B abolished KDM3A/B enrichments on the *AXIN2* promoter in ALDH$^+$/EpCAM$^+$ cells isolated from PS1. **b.** ChIP assays confirmed that the knockdown of KDM3A/B increased the levels of H3K9me2 on the *AXIN2* promoter in ALDH$^+$/EpCAM$^+$ cells isolated from PS1. **c.** ChIP assays showed that the knockdown of KDM3A/B did not affect β-catenin binding on the *AXIN2* promoter in ALDH$^+$/EpCAM$^+$ cells isolated from PS1. **d.** ChIP assays confirmed that the knockdown of KDM3A/B abolished KDM3A/B enrichments on the *DKK1* promoter in ALDH$^+$/EpCAM$^+$ cells isolated from PS1. **e.** ChIP assays confirmed that the knockdown of KDM3A/B increased the levels of H3K9me2 on the *DKK1* promoter in ALDH$^+$/EpCAM$^+$ cells isolated from PS1. **f.** ChIP assays showed that the knockdown of KDM3A/B did not affect β-catenin binding on the *DKK1* promoter in ALDH$^+$/EpCAM$^+$ cells isolated from PS1. **P*<0.05, ***P*<0.01, unpaired 2-tailed Student 's *t*-Test (n=3). **g.** Co-IP showed that MLL1 interacted with Flag-KDM3A and HA-KDM3B in 293T cells. **h.** Co-IP showed that MLL1 interacted with endogenous KDM3A/B in HCT116 cells.

Supplementary Figure 9. Uncropped images for immunoblots.

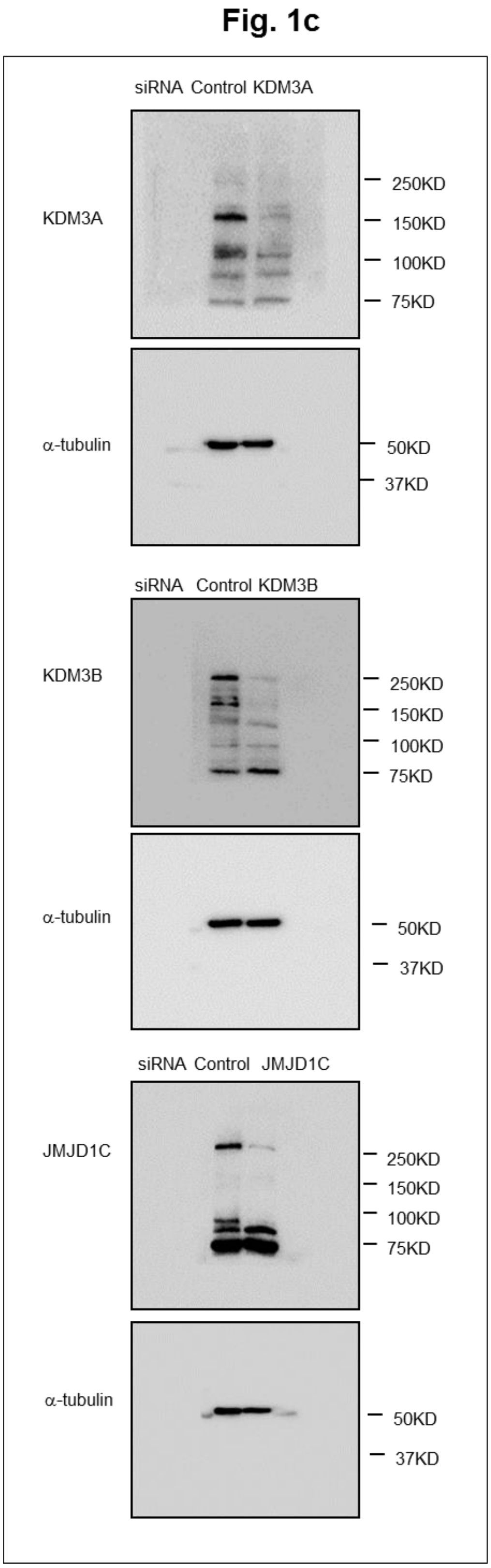

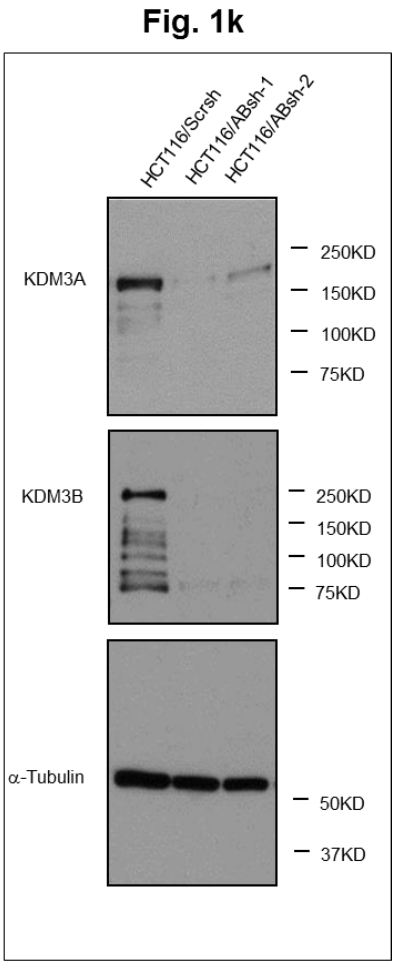

Fig. 2a

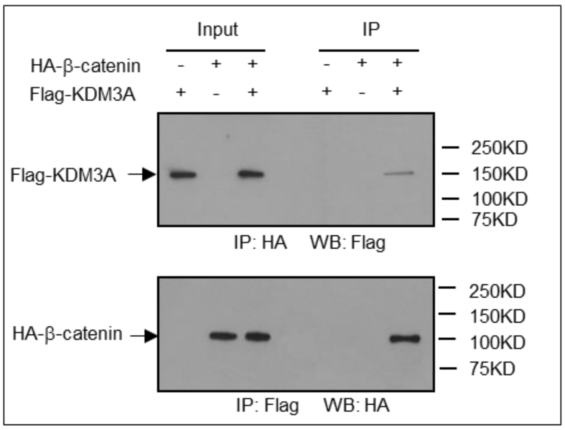

Fig. 2b

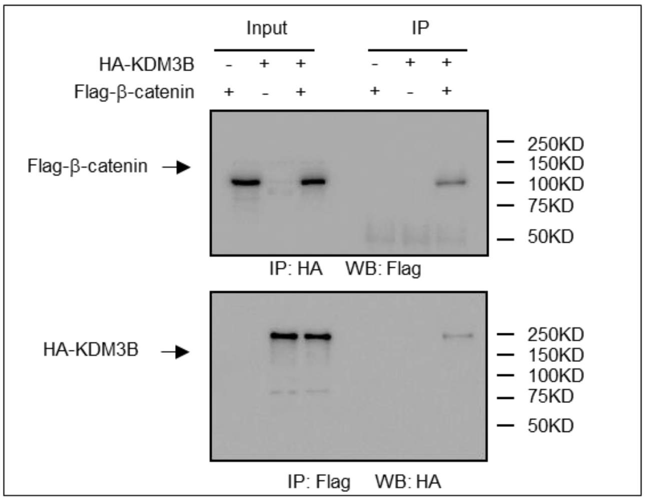

Fig 2c

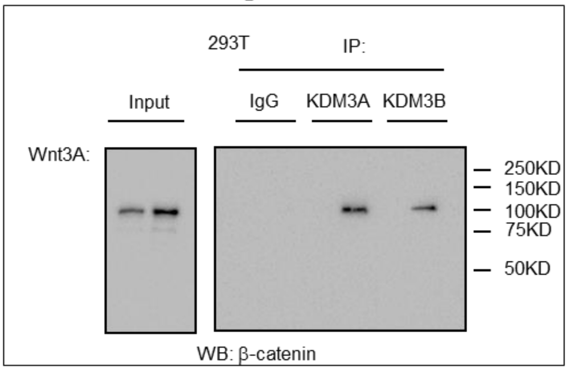

Fig. 2d

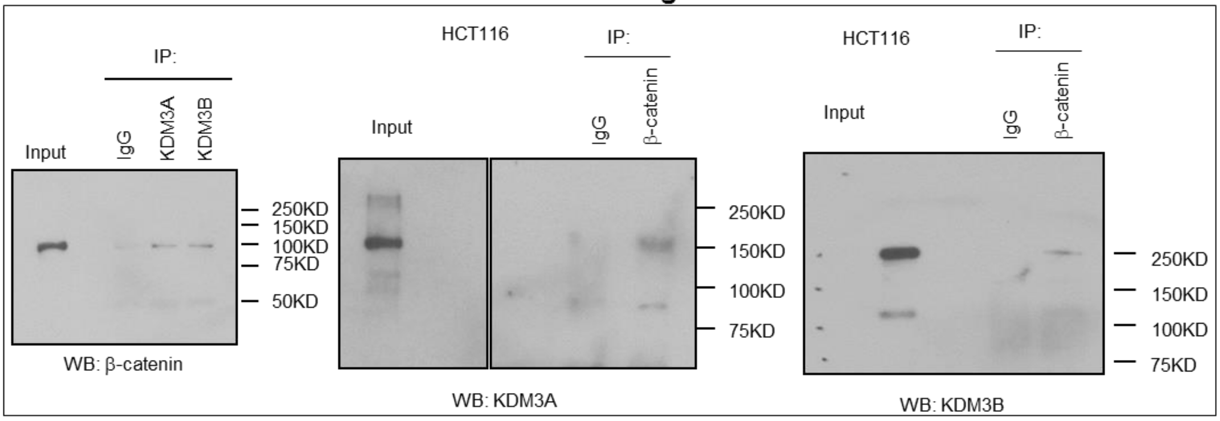

Fig. 2e

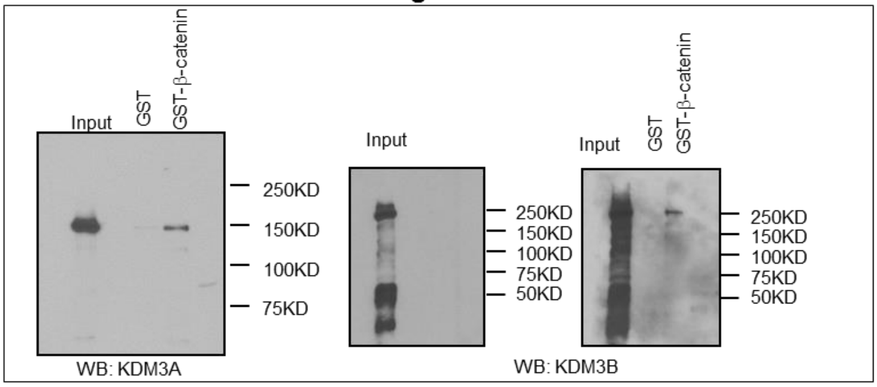

Fig. 3a

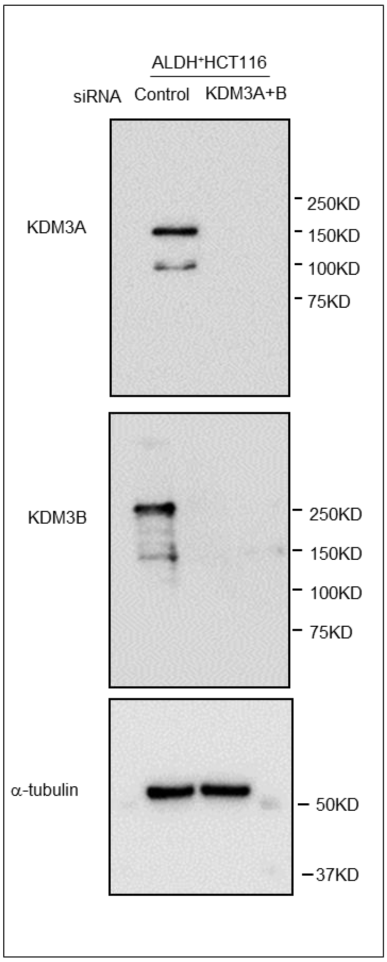

Fig. 4d

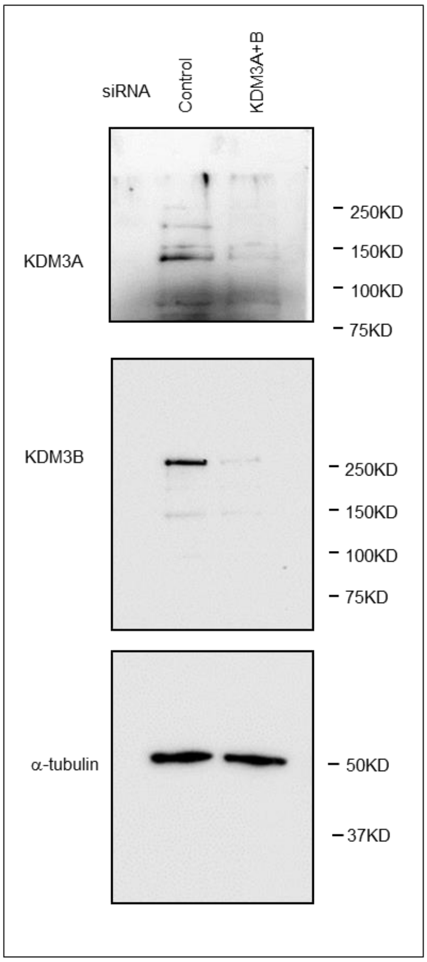

Fig. 4l

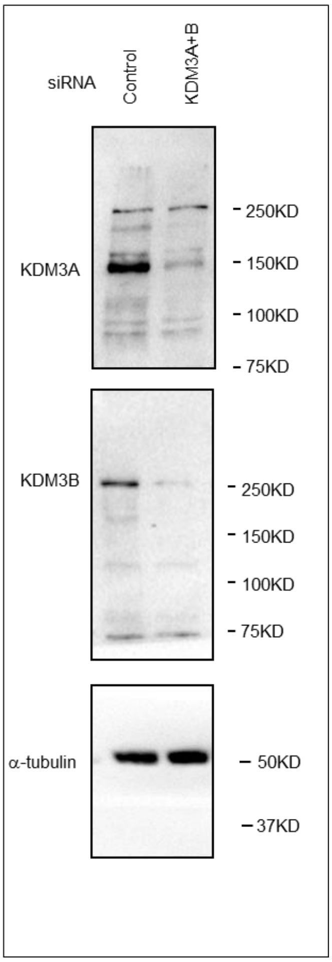

Fig. 5c

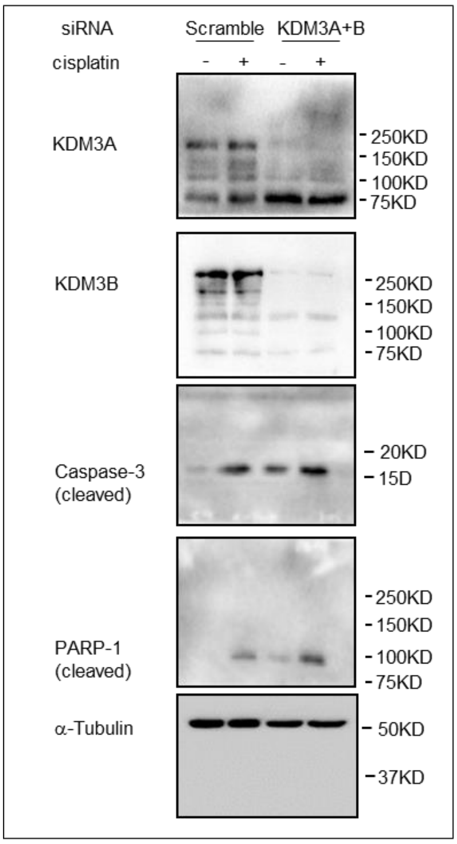

Fig. 5d

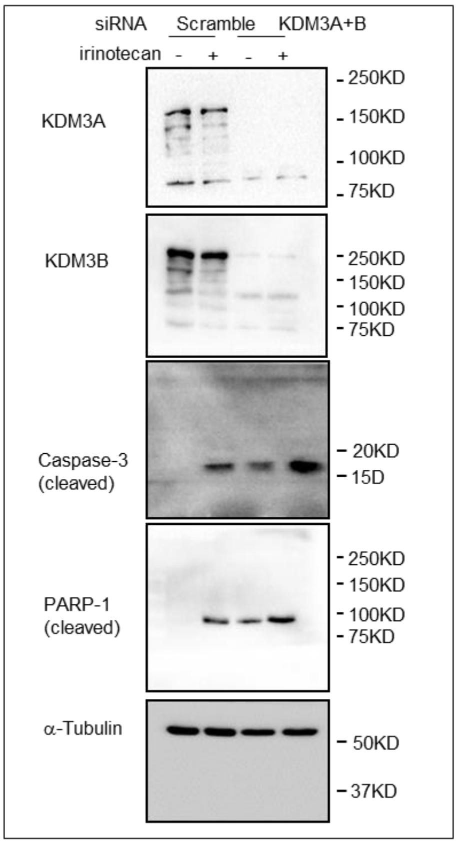

Fig. 5g

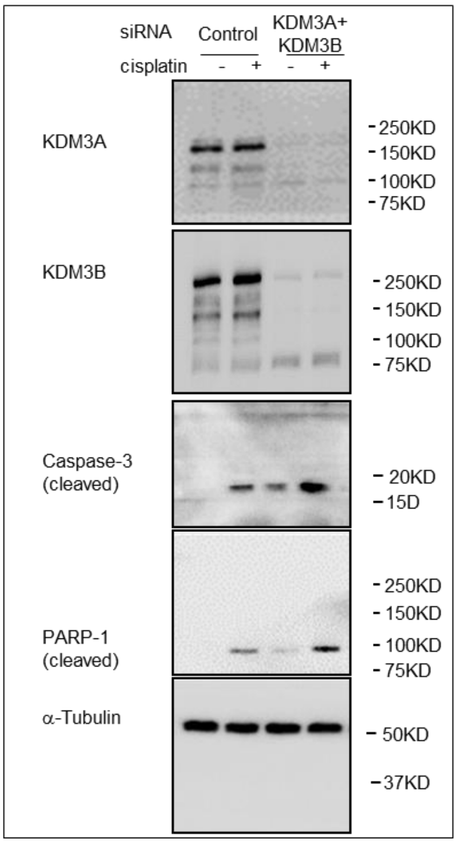

Fig. 5h

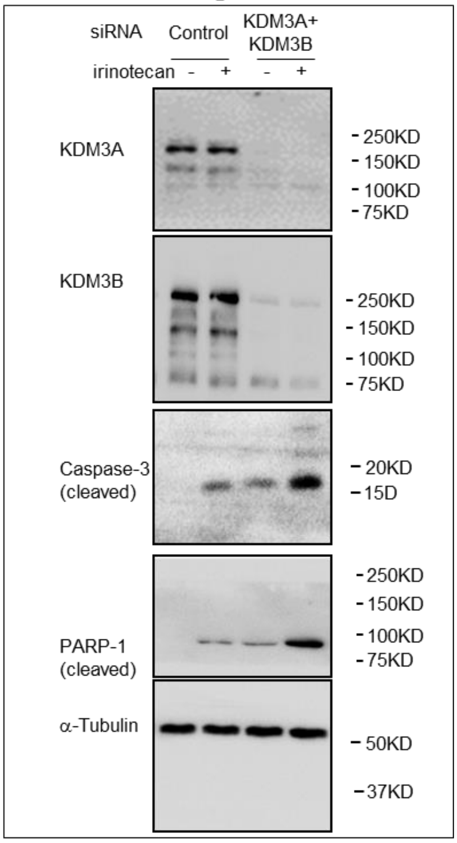

Supplementary Figure 2b

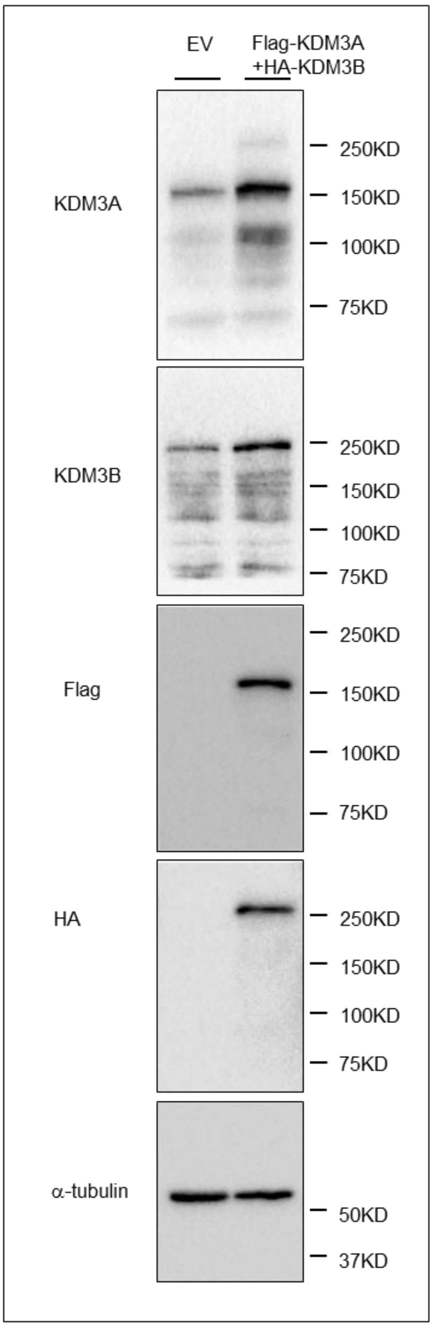

Supplementary Figure 2d

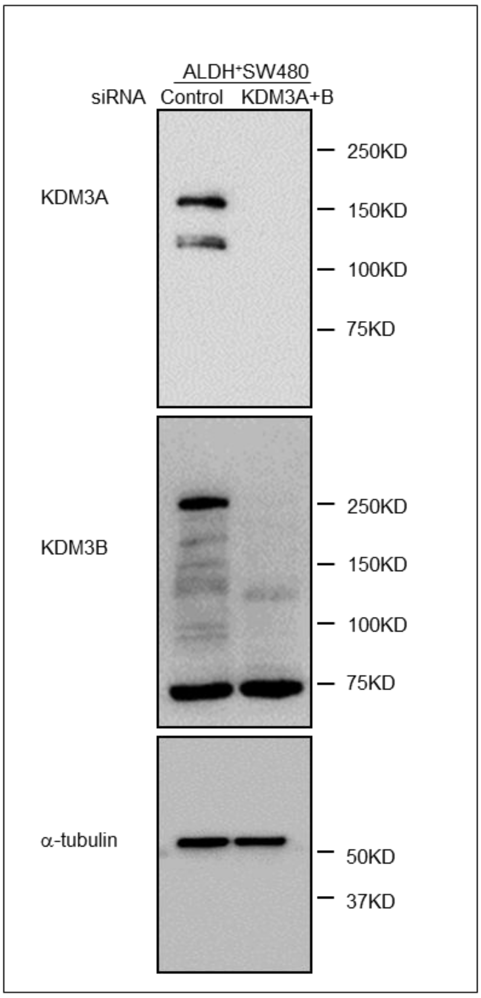

Supplementary Figure 3h

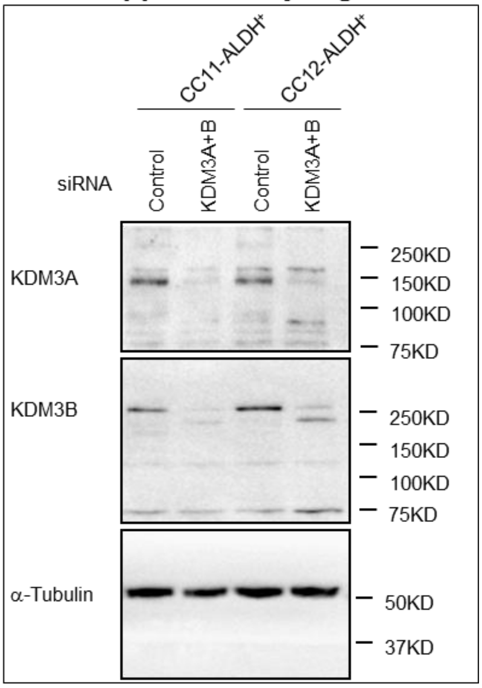

Supplementary Figure 4c

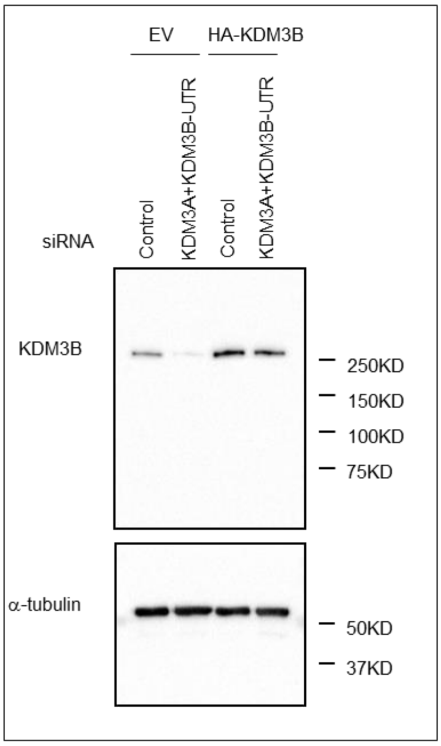

Supplementary Figure 8g

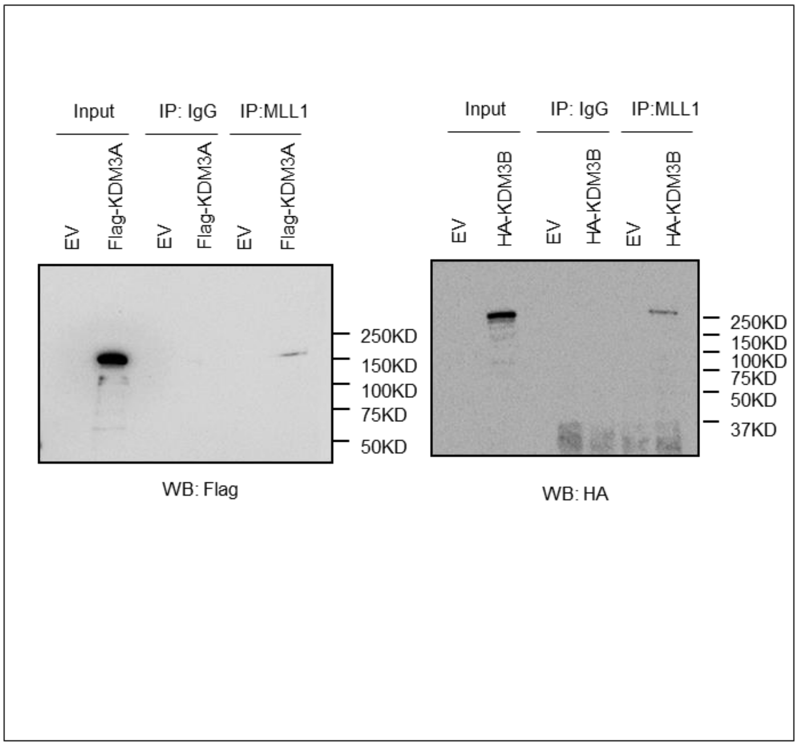

Supplementary Figure 8h

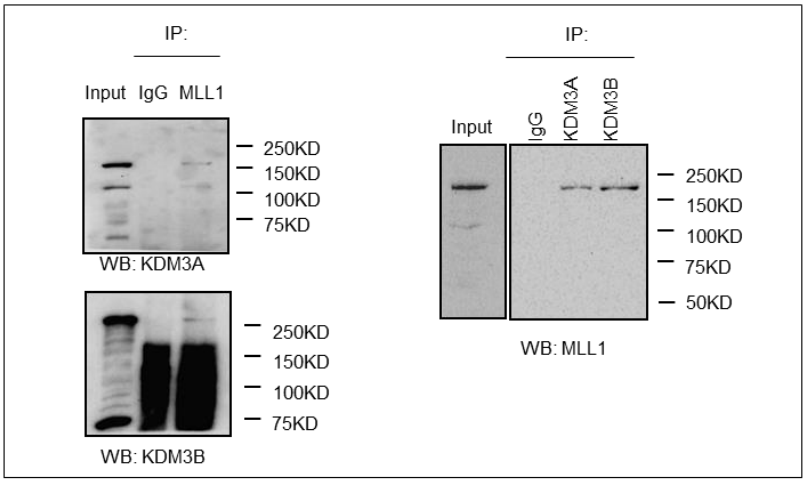

Supplementary Table 1. KDM3A/B are highly expressed in human CRCs.

		KDM3A	KDM3B
Tumor vs Normal	Negative Ranks	1[a]	1[a]
	Positive Ranks	37[b]	34[b]
	Ties	12[c]	15[c]
	Total	50	50
	P Value	*P*<0.001	*P*<0.001

a. CRC < Normal, b. CRC > Normal, c. CRC = Normal

Human colorectal cancers (CRC, *n*=50) and adjacent normal tissues (Normal, *n*=50) were stained for KDM3A/B. The staining intensity was scored as follow: 0, negative staining; + week staining; ++, moderate staining; +++ strong staining. Wilcoxon singed ranks test, *P*<0.001.

Supplementary Table 2. Wnt target genes used for promoter analysis.

Gene Name	Gene ID
MYC	4609
ATOH1	474
AXIN2	8313
BIRC5	332
BMP4	652
CCND1	595
CD44	960
CLDN1	9076
DKK1	22943
EDN1	1906
EFNB1	1947
EFNB2	1948
FGF18	8817
FGF20	26281
FOSL1	8061
GAST	2520
ID2	3398
JAG1	182
JUN	3725
L1CAM	3897
LEF1	51176
MET	4233
MMP7	4316
MSL1	339287
MYCBP	26292
NRCAM	4897
PLAUR	5329
PPARD	5467
TCF4	6925
TCF7	6932
TIAM1	7074

Supplementary Table 3. The primers used for ChIP-qPCR.

Primer Name	Directions	Sequence
AXIN2 WRE	Forward	TTAAATCCACAGCGCAGTTT
	Reversed	AGGTCCTGTTTCCAGCAGTC
AXIN2 A1	Forward	CGTTCAGCCCCTACCCTTCTTA
	Reversed	TGTGCCAAGAATCCCAAACTCT
AXIN2 A2	Forward	GGTGGACCAAGTCCTTACACTCCT
	Reversed	GCATTTCTCCCTCTCCAGGAAA
AXIN2 A3	Forward	TTCCACAGCCATGCACCTCTGAGA
	Reversed	TCATGACCAACCGGTCCTATTCA
AXIN2 A4	Forward	GTATCCACTTTGTCTACTGCTCTC
	Reversed	ACTATGTGGTGGTCTGTGCAATC
AXIN2 A5	Forward	CCACCTTTGCAGCTGAGCTGATCT
	Reversed	ATTACCTCTCGGATCTGCTGCAG
AXIN2 NEG	Forward	GATCTGGCCAATTAGTAGAGCTT
	Reversed	CCCAGGTGTGACTCTCATTGGA
DKK1 WRE	Forward	CAGATCTGCAAACTGCGACT
	Reversed	CTCTAGCAAGACGCCTCTGA
DKK1 D1	Forward	AGCCGGATGAATGTGCTGAGCA
	Reversed	TGAGCGAAGATCAAACGGCCTG
DKK1 NEG	Forward	CCGCCTTGCATGTCTATGTCA
	Reversed	TGCACTGCACAATTTCACAT
CCND1 WRE	Forward	CCATTCTCTGCCGGGCTTTGAT
	Reversed	GGCTCCAGGACTTTGCAACTTC
CCND1 NEG	Forward	CTATCCCAACAACAGACCCT
	Reversed	CAGTATCTGTGCTGCCCATC
MYC WRE	Forward	TTGATCAGAATCGATGCATTT
	Reversed	GGACAAACCGGACGTTTAAT
MYC M1	Forward	TGGTCTTCCCCTACCCTCTCAA
	Reversed	AGGAGAGCAGAGAATCCGAGGA
MYC M1	Forward	AGCGAGGGTAAGAACTCCAA
	Reversed	TTAGAACGTCGATGCTTTCG
